# Final adult height in long-term growth hormone-treated achondroplasia patients

**DOI:** 10.1007/s00431-017-2923-y

**Published:** 2017-05-13

**Authors:** Daisuke Harada, Noriyuki Namba, Yuki Hanioka, Kaoru Ueyama, Natsuko Sakamoto, Yukako Nakano, Masafumi Izui, Yuiko Nagamatsu, Hiroko Kashiwagi, Miho Yamamuro, Yoshihito Ishiura, Ayako Ogitani, Yoshiki Seino

**Affiliations:** 1 Department of Pediatrics, Osaka Hospital, Japan Community Healthcare Organization (JCHO), 4-2-78, Fukushima, Fukushima-ku, Osaka, Osaka 553-0003 Japan; 2 Neonatal Intensive Care Unit, Nara Prefecture General Medical Center, Nara, Japan

**Keywords:** Achondroplasia, Growth hormone, Limb lengthening, Gonadal suppression, Final height

## Abstract

The objective of this study was to evaluate the gain in final height of achondroplasia (ACH) patients with long-term growth hormone (GH) treatment. We analyzed medical data of 22 adult patients (8 males and 14 females) treated with GH at a dose of 0.05 mg/kg/day. Optionally, tibial lengthening (TL) was performed with the Ilizalov method in 15 patients and TL as well as femoral lengthening (FL) in 6 patients. Concomitant gonadal suppression therapy with buserelin acetate was applied in 13 patients. The mean treatment periods with GH were 10.7 ± 4.0 and 9.3 ± 2.5 years for males and females, respectively. GH treatment augmented the final height +0.60 ± 0.52 SD (+3.5 cm) and +0.51 ± 1.29 SD (+2.8 cm) in males and females compared to non-treated ACH patients, respectively. Final height of ACH patients that underwent GH and TL increased +1.72 ± 0.72 SD (+10.0 cm) and +1.95 ± 1.34 SD (+9.8 cm) in males and females, respectively. GH, TL, and FL increased their final height +2.97 SD (+17.2 cm) and +3.41 ± 1.63 SD (+17.3 cm) in males and females, respectively. Gonadal suppression therapy had no impact on final height.

*Conclusions*: Long-term GH treatment contributes to 2.6 and 2.1% of final adult height in male and female ACH patients, respectively.

**Table:** 

**What is Known:** • *ACH is a common form of rhizomelic dwarfism, with an average adult height of 130.4 cm for males and 124.0 cm for females in the Japanese population*. • *Short-term GH improves height standard deviation (SD) scores in ACH patients*.
**What is New:** • *Long-term GH treatment increased the final height of ACH patients +0.60 ± 0.52 SD (+3.5 cm) and +0.51 ± 1.29 SD (+2.8 cm) for males and females, respectively*. • *Average final height SD score increased +1.85 SD with GH and tibial lengthening and +3.27 SD with GH, tibial, and femoral lengthening*.

## Introduction

Achondroplasia (ACH, MIM no. 100800) is the most common form of congenital rhizomelic dwarfism characterized by severe and disproportionate short stature, macrocephaly with a prominent forehead, midface hypoplasia, and trident hands. ACH is inherited as an autosomal dominant trait and is caused by gain of function mutations in the fibroblast growth factor receptor 3 (*FGFR3*) gene. The final height may be as short as 118–145 cm for males and 112–136 cm for females in Caucasian patients [1]. In Japanese patients, the average final height without treatment has been reported as 130.4 cm for males (−7.0 standard deviations [SD] compared with normal Japanese children) and 124.0 cm (−6.4 SD) for females [2].

Classically, limb lengthening has been performed to ameliorate body disproportion [3]. While individual studies suggest that tibial lengthening (TL), femoral lengthening (FL), and combined TL and FL add 4.0–10.5, 3.0–13.0, and 9.0–24.0 cm, respectively, to bone length or standing height of patients with ACH [4–7], a recent systematic review shows that the overall height gain in ACH/hypochondroplasia patients is 6–12 cm [8]. In addition, short-term growth hormone (GH) treatment has also been shown to be effective for accelerating height velocity and improving height SD score in ACH patients [9–12]. GH treatment has been approved only in Japan since 1997. Although nearly 20 years have passed since its approval, there have been no reports on the long-term effects of GH treatment in patients with ACH. Moreover, in recent years, height-targeting novel therapies for ACH have been proposed by various groups [13–15]. Given the exceptional safety record of GH, it is essential that height gain achieved by these treatments is not inferior to that of GH. We therefore investigated the effect of long-term GH treatment in patients with ACH.

## Subjects and methods

### Study design

We conducted a retrospective cohort study regarding the effect of long-term GH treatment in ACH patients. All patients were treated with subcutaneous GH injections. Some received limb lengthening and/or gonadal suppression therapy (comprehensive treatment) as well. The primary outcome was gain in final height due to GH. The secondary outcome was increase in final height with comprehensive treatment.

### Patients

**Fig. 1:**
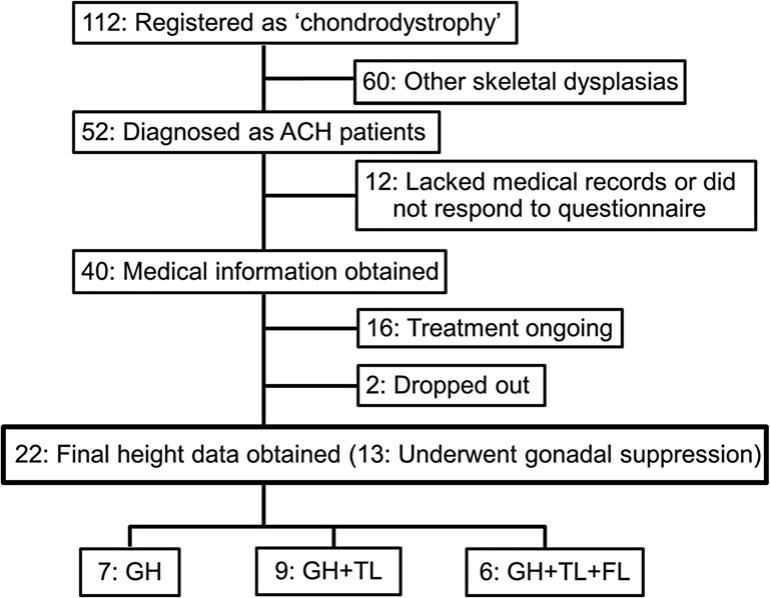
Enrollment and follow-up. One hundred twelve patients were registered as “chondrodystrophy” in our institution. Sixty patients with hypochondroplasia and other skeletal dysplasias were excluded. A total of 52 ACH patients were enrolled in this study. No clinical data of 12 patients were available. Medical information of 40 patients with ACH who had received GH treatment was obtained through medical records and/or questionnaires. GH treatment is ongoing in 16 patients and 2 patients had discontinued GH treatment due to severe deformation of their spine and lower extremity. Twenty-two patients (8 males and 14 females) had reached final height and were included in the analysis. Pretreatment data of two males and one female were partially missing. Among these 22 patients, seven (three males and four females) were treated only with GH, nine (four males and five females) underwent TL in addition to GH, and six (one male and five females) underwent FL in addition to GH and TL. Concomitant gonadal suppression therapy was performed in 13 patients (3 males and 10 females). *GH* growth hormone, *ACH* achondroplasia, *TL* tibial lengthening, *FL* femoral lengthening

**Fig. 2:**
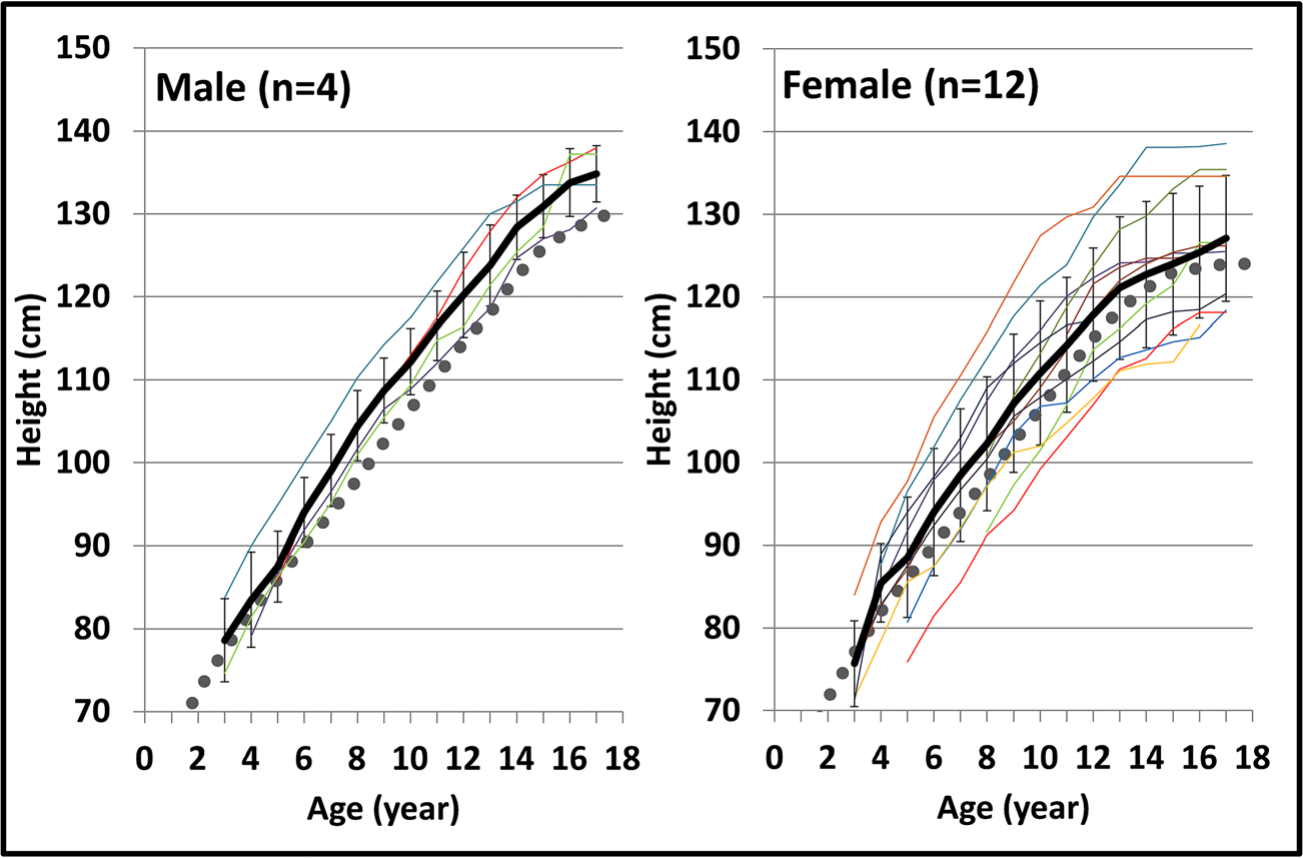
GH treatment improves final height in ACH patients. Growth curves of each patient with ACH who had long-term GH treatment. The *black lines* indicate the mean ± SD of the patients. The *gray dotted lines* show the average curves of non-treated patients with ACH according to reference 2. Following GH treatment, 69% (11/16) of the patients maintained height above average. Since the ages when GH was started differ among patients, the mean heights at age 3 years do not match the height SD scores at the start of the treatment in Table 1. *GH* growth hormone, *ACH* achondroplasia

**Fig. 3:**
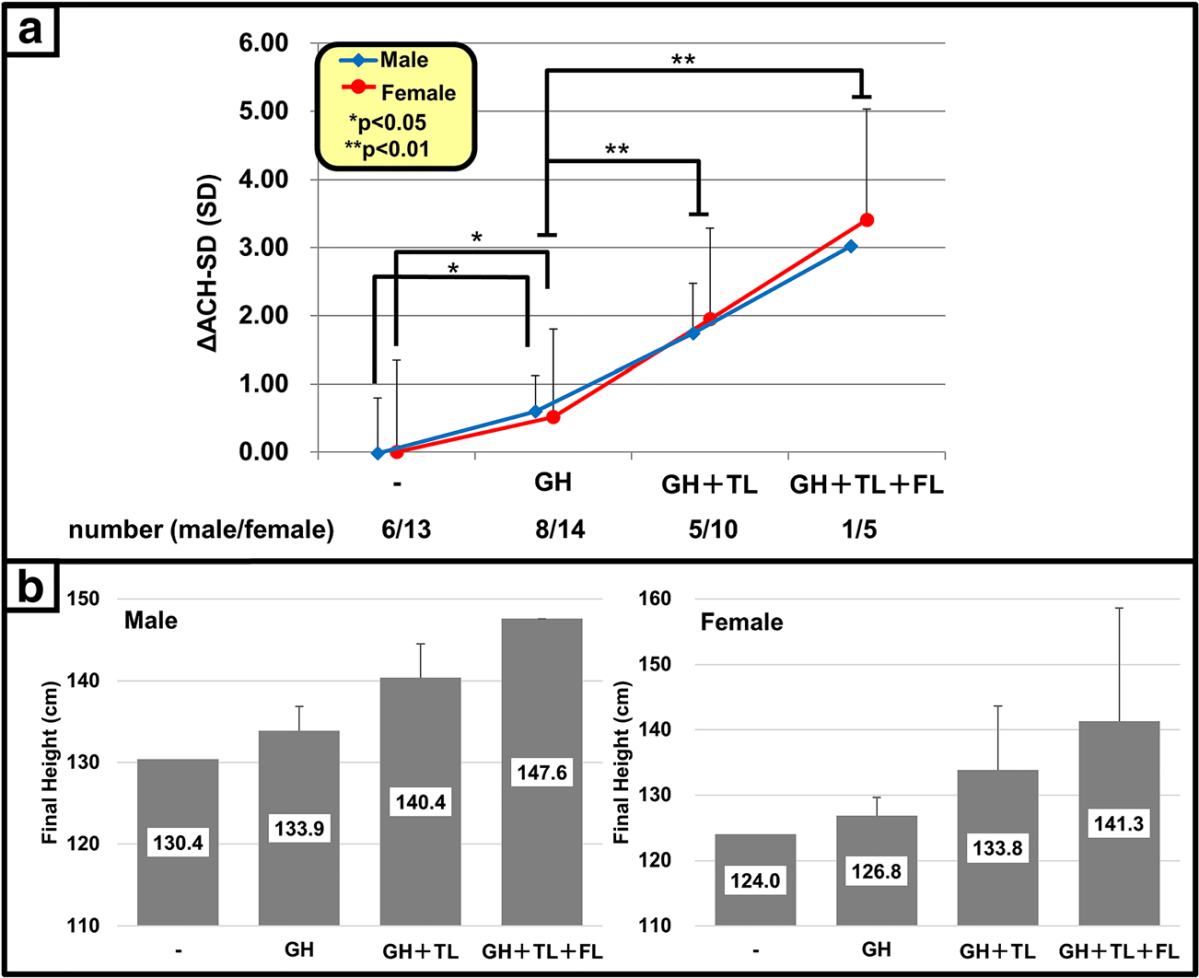
Overall effect of comprehensive treatment. **a** Comparison of ACH-SD scores (∆ACH-SD). GH treatment alone (*n* = 22) increased ACH-SD scores by +0.60 ± 0.52 SD for males (*p* < 0.05) and +0.51 ± 1.29 SD for females (*p* < 0.05). Final height ACH-SD scores attained with the combination of GH and TL (*n* = 15) were +1.85 ± 1.16 SD (*p* < 0.01) (+1.72 ± 0.72 SD and +1.95 ± 1.34 SD for males and females, respectively). The combination of GH, TL, and FL (*n* = 6) increased final height ACH-SD scores +3.27 ± 1.46 SD (*p* < 0.01) (+2.97 SD and +3.41 ± 1.63 SD for males and females, respectively). **b** Calculated final height based on ∆ACH-SD scores. *GH* growth hormone, *ACH* achondroplasia, *TL* tibial lengthening, *FL* femoral lengthening

Fifty-two patients with ACH (23 males and 29 females) were enrolled in this study (Fig. 1). All patients had visited our hospital for treatment and were clinically diagnosed as ACH due to severe rhizomelic short stature, characteristic facial features, and trident hands. Bone X-ray features of all of the patients included thick and short longitudinal bones, metaphyseal cupping, narrowing of the lumber interpediculate distance, and narrow ischiatic notches. When the clinical diagnosis was uncertain, *FGFR3* gene analysis was performed by direct sequencing. The typical p.Gly380Arg mutation was detected in all tested patients. Medical records and/or questionnaires from 40 patients with ACH (17 males and 23 females) that underwent GH treatment were obtained. Twenty-two patients (8 males and 14 females) had reached final height and were included in the analysis. Of the 22 ACH patients, gene analysis was performed in 10 patients (45%). Annual height throughout GH treatment was available in only 16 patients (70%, 4 males and 12 females) (Fig. 2). Data at initiation of GH treatment was accessible in 19 patients (86%, 6 males and 13 females) (Fig. 3).

### Follow-up protocol

Recombinant human GH was injected subcutaneously at a dose of 0.05 mg/kg/day. Physical examination and blood/biochemical examinations were performed every 3–4 months. Limb lengthening and/or gonadal suppression therapy were added according to the expectations of the patients and/or parents. Limb lengthening was performed by the Ilizalov method [16]. For gonadal suppression therapy, a daily dose of 0.9 mg buserelin acetate was provided as a nasal spray when patients entered puberty.

On every visit, standing height was measured by a single well-calibrated stadiometer. To assess the effect of limb lengthening, we measured two values. One was the actual reading of the Ilizarov external fixator scale. We termed this value “theoretical lengthening value (TLV).” The other, “standing height gain (SHG),” was calculated by subtracting the pre-lengthening height from the post-lengthening height. In this study, we focused on SHG as the actual limb lengthening value. We subtracted SHG from final height to determine growth due to GH alone.

### Statistical analysis

Height SD scores were compared with normal Japanese children (no secular trend since 1990) of the same sex and age. To further highlight treatment efficacy, SD scores were also calculated using the average height of non-treated patients with ACH derived from a nationwide survey of 135 ACH patients from 1991 to 1995 [2]. SDs of normal Japanese children were adopted for this purpose because SDs of non-treated Japanese ACH patients are not available. We refer to this score as ACH-specific SD score (ACH-SD).

Statistical analyses were performed by the Mann–Whitney *U* test and Pearson’s correlation coefficient using SPSS software V23.0 (IBM Japan, Tokyo). *P* values <0.05 were considered significant.

## Results

### Final height attained with GH treatment

Table 1 shows the background of the patients. Mean age and height SD scores did not differ significantly between males and females. The mean treatment period and age at termination were 10.7 ± 4.0 and 16.2 ± 1.3 years for males and 9.3 ± 2.5 and 14.7 ± 1.8 years for females, respectively. Growth records of 16 patients with GH treatment show that 69% of patients (4/4 and 8/12 in males and females, respectively) maintained their height above the average curve of non-treated ACH patients (Fig. 2). Consequently, the increase in ACH-SD score (∆ACH-SD) with GH treatment at final height was +0.60 ± 0.52 SD for males (*p* < 0.05) and +0.51 ± 1.29 SD for females (*p* < 0.05) (Fig. 3a). The average final height attained by GH treatment was calculated as 133.9 cm (+3.5 cm) and 126.8 cm (+2.8 cm) for males and females, respectively (Fig. 3b).

**Table 1:** Background of the patients

	Male	Female
Number of patients	8	14
Age at start of treatment (years)	5.2 ± 3.9 (3.0 to 14.0)	5.5 ± 2.7 (3.0 to 11.0)
Height SD score at GH initiation (SD)	−5.11 ± 0.84 (−5.89 to −4.46)	−5.22 ± 1.33 (−7.16 to −3.42)
Height ACH-SD score at GH initiation (SD)	0.05 ± 0.80 (−1.43 to 1.40)	−0.28 ± 1.35 (−2.35 to 1.94)

### Final height attained with comprehensive treatment (GH and limb lengthening)

TL (*n* = 15) and FL (*n* = 6) were performed at ages 12.4 ± 3.2 years (range, 9.0–17.0 years) and 12.6 ± 2.4 years (range, 10.0–17.0 years), respectively. The mean TLV and SHG are indicated in Table 2. Although TLV and SHG were not exactly consistent, there was no statistically significant difference between TLV and SHG.

**Table 2:** Effect of limb lengthening

		No. of patients	TLV** (cm)**	SHG** (cm)**
Tibial lengthening	Males	5	8.1 ± 1.6 (6.4 to 10.2)	6.9 ± 2.4 (5.1 to 10.2)
	Females	10	8.2 ± 2.2 (3.1 to 10.0)	8.9 ± 2.3 (5.0 to 13.0)
	Total	15	8.2 ± 2.0	8.3 ± 2.4
Femoral lengthening	Males	1	10.7	7.0
	Females	5	8.6 ± 1.1 (8.5 to 10.0)	6.9 ± 2.1 (3.6 to 9.0)
	Total	6	9.0 ± 1.3	7.0 ± 1.9

Values are mean ± SD (range)

*TLV* theoretical lengthening value, *SHG* standing height gain

Comprehensive treatment with GH and limb lengthening significantly increased final height in ACH patients (Fig. 3a). The ∆ACH-SD with GH and TL was +1.72 ± 0.72 SD and +1.95 ± 1.34 SD for males and females, respectively. The mean of ∆ACH-SD with GH, TL, and FL was +2.97 SD and +3.41 ± 1.63 SD for males and females, respectively. According to the ∆ACH-SD scores, the average final heights of male and female patients were calculated as 140.4 cm (+10.0 cm) and 133.8 cm (+9.8 cm) with GH and TL and 147.6 cm (+17.2 cm) and 141.3 cm (+17.3 cm) with GH, TL, and FL, respectively (Fig. 3b). There was no significant correlation between lengthening values and age or height at surgery.

### Gonadal suppression and other considerations

Gonadal suppression was performed in 13 of the 22 patients (59%, Table 3). The mean height ACH-SD score at GH initiation tended to be lower in patients who received this therapy than in patients who did not. Buserelin acetate did not statistically increase final height of ACH patients (*p* = 0.33).

**Table 3:** Effect of gonadal suppression therapy in addition to GH treatment

Gonadal suppression therapy	-	+
Number of patients	9	13
ACH-SD score at GH initiation	0.12 ± 1.23 (−1.60 to 1.46)	−0.32 ± 1.20 (−2.35 to 1.94)
ACH-SD score at final height	0.51 ± 1.17 (−0.89 to 2.75)	0.29 ± 1.08 (−1.40 to 2.15)
Height ΔACH-SD score (SD)	0.42 ± 0.67 (−0.57 to 1.29)	0.58 ± 0.86 (−0.40 to 2.05)

Values are mean ± SD (range)

No patients received protein anabolic hormones and/or other treatments for short stature. No patients had major adverse events or worsening of complications, even after long-term GH treatment.

## Discussion

GH treatment for ACH patients has been approved in Japan with an eligibility criteria of (1) height shorter than −3.0 SD and (2) age older than 3 years. In some patients, GH treatment was started at ages much older than 3 years because they were recruited into clinical trials before the drug was approved. Since ACH patients usually have little pubertal growth spurt, calculating SD scores from the mean height of normal children will attenuate the post-pubertal scores by 2 SD. Accordingly, we assessed final height using the ACH-SD score. Although there was a difference in pretreatment height ACH-SD between males and females, it ranged within ±0.3 and was not statistically significant. We therefore considered the patients in this study as a representative group.

GH treatment increased the final height ACH-SD score +0.60 SD and +0.51 SD for males and females, respectively. This translates to an addition of 3.5 cm for males and 2.8 cm for females to the final height of non-treated ACH patients. Previous reports have shown that short-term GH treatment in ACH patients increases height SD scores from +0.3 to +1.6 SD during 2 to 6 years of treatment [9–12]. Our data shows that the gain in height SD is mostly conserved until final height. Of note, SHG inevitably includes GH induced growth during TL and FL. Thus, we may have underestimated final height SD attributed to GH treatment despite using ACH-SD.

In Japanese children with GH deficiency, height gain by GH treatment corresponds to 4.1 and 3.1% of final height for males and females, respectively [17]. ACH children gained 2.6 and 2.1% for males and females, respectively. Taking into consideration that ACH is a far severer condition than short stature due to GH deficiency, we consider that the effect of GH treatment is reasonable for this group of patients. However, further investigation concerning GH dose and duration, as well as other treatment options, is necessary.

In our study, the effect of GH varied widely. While ∆ACH-SD scores of six (26%) patients were greater than +1.0 SD with a maximum of +2.84 SD (+16.5 cm), those in another six (26%) patients had scores below 0 SD and the lowest was −0.57 SD (−3.3 cm). Genetic background, treatment compliance, or treatment duration may have contributed to the difference. Some patients tended to be less responsive and reached final height at an age slightly earlier than non-treated patients. Since GH can accelerate bone maturation in idiopathic short stature, further optimization of GH dose or duration may be necessary [18]. When new height targeting treatments that are currently under development become available, it may be reasonable to use GH only in patients that respond well.

Limb lengthening still remains to be the most effective measure to increase final height in ACH patients. However, patients need to overcome many problems, such as stiffness of the Achilles tendon, recurrent fractures, asymmetry of the legs, focal bacterial infection, and pain [5–7, 19]. In this study, we found no correlation between surgical age and lengthening values. Interestingly, we seem to consistently achieve greater height gain with GH and limb lengthening than that achieved by limb lengthening alone [8]. This suggests that GH, at least in part, may contribute to the results, although the expertise of the orthopedic surgeons most obviously plays a major role.

Gonadal suppression was performed in attempt to delay epiphyseal fusion and sustain growth. The effect of gonadal suppression on final adult height is controversial. In our study, ACH-SDs at the initiation of GH treatment in patients that underwent this therapy tended to be lower than those in patients that did not. Although a significant change in final height was not detected in our study, a selection bias (shorter patients) may have been present. It is also possible that buselerin acetate did not suppress gonadotropins as thoroughly as leuprolerin depot injections. Recent studies have shown that more effective reagents (i.e., aromatase inhibitors) in combination with GH may increase height potential in pubertal children [20]. Taken together, there still seems to be room for further exploration especially when newer height targeting therapeutic agents become available.

A limitation of this study is that data were collected retrospectively in one institution without non-treated controls. Some difficulties were encountered while collecting data. Medical records of patients whose last visit was more than a decade ago were not available, the questionnaires were not necessarily completely filled out, and patients who reached their final height comprised 65.7% of all included patients. Due to these limitations, some data were analyzed using a small number of patients. On the other hand, since all patients in this study have been examined and treated at our institution, the advantages are the accuracy and consistency of diagnosis, data collection, and clinical management.

In conclusion, long-term GH treatment contributes to 2.6 and 2.1% of final adult height in male and female ACH patients, respectively, without any severe side effects. Final adult height attained with GH will serve as a benchmark when evaluating novel therapeutic agents under development.

## Abbreviations

ACH: Achondroplasia
ACH-SD: ACH-specific SD score
FGFR3: Fibroblast growth factor receptor 3
FL: Femoral lengthening
GH: Growth hormone
SD: Standard deviations
SHG: Standing height gain
TL: Tibial lengthening
TLV: Theoretical lengthening value
